# Removal of Mn(II) from Acidic Wastewaters Using Graphene Oxide–ZnO Nanocomposites

**DOI:** 10.3390/molecules26092713

**Published:** 2021-05-05

**Authors:** Eduardo Leiva, Camila Tapia, Carolina Rodríguez

**Affiliations:** 1 Departamento de Química Inorgánica, Facultad de Química, Pontificia Universidad Católica de Chile, Santiago 7820436, Chile; camilatapiape@gmail.com (C.T.); cnrodriguez@uc.cl (C.R.); 2 Departamento de Ingeniería Hidráulica y Ambiental, Pontificia Universidad Católica de Chile, Santiago 7820436, Chile; 3 Departamento de Química, Facultad de Ciencias, Universidad de Chile, Ñuñoa 7800003, Chile

**Keywords:** adsorption, manganese, graphene oxide, zinc oxide, nanomaterials, acid mine drainage

## Abstract

Pollution due to acidic and metal-enriched waters affects the quality of surface and groundwater resources, limiting their uses for various purposes. Particularly, manganese pollution has attracted attention due to its impact on human health and its negative effects on ecosystems. Applications of nanomaterials such as graphene oxide (GO) have emerged as potential candidates for removing complex contaminants. In this study, we present the preliminary results of the removal of Mn(II) ions from acidic waters by using GO functionalized with zinc oxide nanoparticles (ZnO). Batch adsorption experiments were performed under two different acidity conditions (pH1 = 5.0 and pH2 = 4.0), in order to evaluate the impact of acid pH on the adsorption capacity. We observed that the adsorption of Mn(II) was independent of the pH_PZC_ value of the nanoadsorbents. The qmax with GO/ZnO nanocomposites was 5.6 mg/g (34.1% removal) at pH = 5.0, while with more acidic conditions (pH = 4.0) it reached 12.6 mg/g (61.2% removal). In turn, the results show that GO/ZnO nanocomposites were more efficient to remove Mn(II) compared with non-functionalized GO under the pH2 condition (pH2 = 4.0). Both Langmuir and Freundlich models fit well with the adsorption process, suggesting that both mechanisms are involved in the removal of Mn(II) with GO and GO/ZnO nanocomposites. Furthermore, adsorption isotherms were efficiently modeled with the pseudo-second-order kinetic model. These results indicate that the removal of Mn(II) by GO/ZnO is strongly influenced by the pH of the solution, and the decoration with ZnO significantly increases the adsorption capacity of Mn(II) ions. These findings can provide valuable information for optimizing the design and configuration of wastewater treatment technologies based on GO nanomaterials for the removal of Mn(II) from natural and industrial waters.

## 1. Introduction

The progressive scarcity of water resources is among the major concerns worldwide [1,2,3]. The lower water availability produced by the marked reduction in rainfall and water pollution has a considerable impact on several areas of the world [2]. Specifically, heavy metal contamination significantly affects water quality, due to its persistence, non-degradation, and toxicity at low concentrations [4,5]. Heavy metals can be released from natural sources, such as volcanic activities, or through anthropogenic sources, such as mining operations, because of the generation of acid mine drainage (AMD) runoffs [4,6,7]. These pollutants in AMD waters have adverse effects on the ecosystem, contaminating surface and groundwater resources in various regions [8,9]. For this reason, the development of technological alternatives for the treatment and remediation of metal-enriched waters has become a key challenge to reduce the negative environmental impacts on water resources.

In recent years, the removal of Mn(II) from contaminated waters has received great interest. Manganese can occur in five different oxidation states, namely, Mn(II), Mn(III), Mn(IV), Mn(VI) and Mn(VII); however, the divalent form (Mn(II)) predominates in most contaminated waters [10]. The intake of high concentrations of Mn can cause several toxic effects, such as sexual impotence, damage to the human central nervous system and endocrine system, growth retardation and muscle fatigue [11,12,13]. Additionally, at the metabolic level, it has been seen that Mn concentrations can cause peroxidative damage in fish tissues [14]. Consequently, the presence of Mn in natural waters is not desirable since, depending on the concentration, it can be potentially toxic.

Several approaches for heavy metal removal have been employed, such as ion exchange, precipitation, reverse osmosis, adsorption, membrane separation, bioremediation and phytoremediation [15,16,17,18,19,20,21,22]. Likewise, the removal of metallic pollutants and the degradation of persistent organic pollutants has also been explored by different technologies based on photocatalyst applications [23,24,25]. Among these methods, adsorption processes have been commonly used for metal removal, mainly due to their versatility of application, design flexibility, potential regeneration of adsorbents, cost-effectiveness, lower secondary contamination and high efficiency [15,19,26]. The adsorbents used are variable, including low-cost compounds, such as biochar, activated carbon or clays, or more advanced compounds, such as polymers, metal oxides and nanomaterials [27,28,29,30,31,32,33,34,35]. However, many of the absorbents in some cases have low adsorption capacities and a smaller specific area, which makes it difficult to remove heavy metals. Based on this disadvantage, the application of nanomaterials may be desirable given their improved properties, such as their greater surface area, functionalization potential and higher chemical reactivity, which facilitate the adsorption of heavy metals.

Graphene oxide (GO) is among the most studied nanomaterials for the removal of heavy metals [36,37]. GO has a large specific area (2630 m^2^/g) that favors interaction with heavy metals and at the same time is susceptible to functionalization with different oxygen-containing functional groups, such as carboxyl and hydroxyl groups, that can considerably improve the adsorption of certain heavy metals. In the same way, the interactions between the lamins by π-π stacking and van der walls forces favor the adsorption capacity of heavy metals [36,38]. In fact, some studies have reported the potential of GO for the adsorption of Mn(II). Yang et al. [39] demonstrated that the adsorption of Mn(II) from aqueous solution by Sodium Alginate/Graphene Oxide Composite Double-Network Hydrogel Beads was optimal at pH 6.0. Furthermore, they observed that after seven adsorption–desorption cycles, the adsorption capacity remained unchanged at 18.11 mg/g, which supports that GO and its functionalizations may be a good candidate for the removal of Mn(II). Additionally, Xu et al. [40] showed that a poly (sodium acrylate)-graphene oxide (PSA-GO) double network hydrogel adsorbent was efficient for Mn(II), reaching a maximum adsorption capacity of 165.5 mg/g at pH = 6.0. Likewise, it has been shown that GO functionalized with ZnO nanoparticles is efficient for the removal of various metals, such as Cu, Cd, Co, Cr, Hg and Pb, resulting in considerable improvements in the adsorption capacity of non-functionalized GO [41,42,43]. Recently, our group demonstrated that the functionalization of GO with ZnO nanoparticles improves the adsorption capacity of Cu and to a lesser extent of Al from acidic waters [44]. Despite these advances, no approaches have been developed to evaluate the potential applications of GO and its functionalization in the removal of Mn(II) ions from acidic waters where pH conditions can affect adsorption processes. This is relevant since AMD waters can have high concentrations of Mn(II) and low pH. In addition, the growing attention that Mn(II)-contaminated water courses has received is driving the development of emerging and efficient technologies to remove this contaminant.

Here, we report the preliminary results of the application of GO/ZnO for the removal of Mn(II) under acidic conditions. Experimental data were fitted using Langmuir model isotherms in adsorption experiments considering two different pH conditions (pH1 = 5.0 and pH2 = 4.0). Thus, we describe the performance of GO/ZnO nanocomposites as a potential candidate for their application in the treatment and remediation of Mn(II)-enriched wastewaters.

## 2. Materials and Methods

### 2.1. Materials

All the chemical reagents used in this study were of analytical reagent grade, and the solutions used were prepared with deionized water. For GO preparation, graphite powder was purchased from Merck. ZnO nanoparticles were obtained from Sigma Aldrich and used without further purification. Manganese-enriched synthetic acidic wastewater was prepared by adding NaNO_3_ and MnSO_4_·H_2_O to deionized (DI) water. pH was adjusted with hydrochloric acid (HCl) purchased from Merk, while absolute ethanol (C_2_H_5_OH) used to prepare the nanocomposites (GO/ZnO) was also purchased from Merck.

### 2.2. Preparation of GO and GO/ZnO Nanocomposites

The modified Hummers method [45] was used to prepare GO nanosheets. The GO was prepared from a mixture of graphite powder with additional KMnO_4_. Specifically, the GO was obtained by following the procedure described by Rodríguez et al. [32]. The obtained single-layered GO suspension was oven-dried at 70 °C for 4 h.

Commercial ZnO nanoparticles with an average size of 100 nm were dispersed in absolute ethanol (0.33 mg/mL) and then sonicated for 40 min. Then, the resulting solution of ZnO nanoparticles was slowly added drop by drop to the GO dispersion (2.5 mg/mL) previously prepared. The resulting mixture was washed several times with ethanol and then placed in a water bath to remove the remaining ethanol. Finally, the suspension of GO/ZnO nanocomposites obtained was oven-dried at 70 °C for 4 h for their characterization and use in batch adsorption experiments. 

### 2.3. Characterization Techniques

Infrared spectra were recorded on a Fourier transform infrared (FT-IR) 4600 Spectrometer (JASCO, Easton, PA, USA) in the range of 400–4000 cm^−1^ at a resolution of 1 cm^−1^ using spectroscopic quality KBr powder (KBr pellet method). The specific surface area of the GO and GO/ZnO nanocomposites was calculated by Brunauer–Emmett–Teller (BET) N_2_ adsorption–desorption analysis (Micromeritics Instruments Corp., Norcross, GA, USA). The structure, size and morphology of GO, ZnO and GO/ZnO nanocomposites were examined using a scanning electron microscope (SEM) (JSM-IT300LV, JEOL, Tokyo, Japan) coupled with energy-dispersive X-ray spectroscopy (Oxford Instruments, High Wycombe, UK) (SEM-EDX). The elemental mapping on the nanometer scale of GO, ZnO and GO/ZnO nanocomposites were measured using EDX analysis. Finally, the pH of point of zero charge (pH_PZC_) value was determined following the method described by Rodríguez et al. [32].

### 2.4. Equilibrium Adsorption Study

Batch experiments were carried out in 40 mL tubes to obtain the equilibrium isotherms of Mn(II) with GO and GO/ZnO as nanoadsorbents. The experiments were performed with a variable concentration of Mn(II) ions (added as MnSO_4_·H_2_O) under two pH conditions (pH1 = 5.0 and pH2 = 4.0). pH was adjusted to 4.0 by adding 0.01 M HCl. For each batch, 20 mg of each nanoadsorbent (GO and GO/ZnO) was aggregated in 40 mL tubes, and the residual concentrations of Mn(II) ions were measured after equilibrium time (20 h) in filtered (0.22 µm) samples. The experiments were performed with shaking (380 rpm) at room temperature (22–24 °C). Additionally, solid phases were obtained from the samples by centrifugation. 

The sorption capacity at equilibrium *q_e_* (mg/g sorbent) was calculated using Equation (1):

(1) $$q_{e}=\frac{\left(C_{0}-C_{e}\right)\cdot V}{m}$$

where *C*_0_ is the initial concentration (mg/L), *C_e_* is the aqueous-phase equilibrium metal concentration (mg/L), *V* is the volume of suspension (L) and m is the mass of the adsorbent (g).

The equilibrium adsorption of Mn(II) ions solutions was measured in the dark at room temperature for 20 h for adsorption/desorption equilibrium in 40 mL tubes with a variable initial concentration of Mn(II) (4.9–10.5 mg/L). 

To evaluate the removal of Mn(II) ions from metal solutions, the removal efficiency (*η*) was calculated using Equation (2):

(2) $$\eta \;\left(\%\right)=\left(\frac{C_{0}-C_{t}}{C_{0}}\right)\cdot 100$$

where *C*_0_ and *C_t_* are the concentration of metal ions at the initial and time *t*, respectively.

### 2.5. Adsorption Isotherms 

The experimental data obtained in batch adsorption experiments were fitted using linear Langmuir-2, Freundlich and Tempkin models (Table 1). The sorption capacity q (mg/g sorbent) was obtained using these models. 

The linear form of the Langmuir-2 isotherm is given as Equation (3):

(3) $$\frac{C_{e}}{q_{e}}=\frac{C_{e}}{q_{m}}+\frac{1}{\left(q_{m}K_{L}\right)}$$

where *q_e_* is the maximum amount of the ions adsorbed at equilibrium (mg/g), *q_m_* is the maximum theoretical adsorbed amount at equilibrium (mg/g), *K_L_* is the Langmuir constant related to the energy of adsorption and *C_e_* is the equilibrium concentration of metal in aqueous solution (mg/L). 

The linear form of the Freundlich isotherm is given as Equation (4):

(4) $$\log\left(q_{e}\right)=\log\left(K_{F}\right)+\frac{1}{n\log\left(C_{e}\right)}$$

where *K_F_* is the Freundlich adsorption capacity, *q_e_* is the maximum amount of ions adsorbed at equilibrium (mg/g), 1/*n* is the sorption constant with a value range between 0 and 1 and *C_e_* is the equilibrium concentration of metal in aqueous solution (mg/L) 

Finally, the linear form of the Temkin isotherm is given as Equation (5):

(5) $$q_{e}=B_{T}In(K_{T}C_{e})$$

where *q_e_* is the amount of ions sorbed (mg/L), *B_T_* is the equilibrium concentration of adsorbate (mg/L) and a short form of expression RT/b_t_ (where *R*, *T* and *b_t_* represent the gas constant (8.314 J/mol K), absolute temperature (*K*) and Tempkin isotherm constant), *K_T_* is the Tempkin isotherm equilibrium binding constant (L/g) and *C_e_* is the equilibrium concentration of metal in aqueous solution (mg/L)

### 2.6. Adsorption Kinetics

To examine the mechanisms that control the adsorption processes onto GO and GO/ZnO nanocomposites, the kinetic behavior was investigated using a pseudo-first-order and pseudo-second-order kinetic equations. For kinetic analysis, 28.4 mg/L of Mn(II) concentration and 20 mg of GO and GO/ZnO nanocomposites were added separately in 40 mL tubes. The solutions were continuously stirred at 380 rpm and at room temperature (22–25 °C), and the sampling was carried out at 10 min, 30 min, 1 h, 2 h, 4 h, 18 h and 24 h. 

The pseudo-first-order (PFO) kinetic model is given as Equation (6) according to Ding et al. [49].

(6) $$\log\left(q_{e}-q_{t}\right)=\log q_{e}-\frac{k_{1}}{2.303}t$$

where *k*_1_ is the constant of first-order adsorption in 1/min, *q_e_* is the adsorption capacity at equilibrium (mg/g) and *qt* is the adsorption capacity at the time *t* (mg/g).

The pseudo-second-order (PSO) kinetic model is given as Equation (7) according to Ho and McKay [50].

(7) $$\frac{t}{q_{t}}=\frac{1}{k_{2}q_{e}^{2}}+\frac{t}{q_{e}}$$

where *k*_2_ is the rate constant of second-order adsorption in g/mg min, *q_e_* is the adsorption capacity at equilibrium (mg/g) and *qt* is the adsorption capacity at the time *t* (mg/g).

To evaluate if the kinetic model is applicable to experimental data, the equilibrium adsorption capacity was determined from the slope and the intercept of straight-line plots of Equations (6) and (7). Furthermore, to characterize the curve of the pseudo-second-order model, the approaching equilibrium factor (*Rw*) was determined. This factor represents the characteristics of the PSO kinetic curve in an adsorption system and is defined by Equation (8) according to Wu et al. [51].

(8) $$R_{w}=\frac{1}{1+k_{2}q_{e}t_{ref}}$$

where *k*_2_ is the rate constant of second-order adsorption in g/mg min, *q_e_* is the adsorption capacity at equilibrium (mg/g) and $t_{ref}$ is the longest operation time (based on kinetic experiments). 

### 2.7. Chemical Analyses

Mn(II) concentrations in the aqueous solutions of batch adsorption experiments were measured using a UV-vis spectrophotometer (DR3900, Hach, Loveland, CO, USA), while pH (PHC301, Hach, Loveland, CO, USA) was measured using a multimeter (Hq40d Multi, Hach, Loveland, CO, USA). 

### 2.8. Quality Assurance/Quality Control (QA/QC)

Quality Assurance/Quality Control (QA/QC) procedures were carried out continuously to ensure the quality and reproducibility of the results obtained during experimentation. 

The material was washed with Milli-Q water during the experimentation process, and analytical grade chemical reagents were used. Likewise, the instruments were calibrated periodically to ensure the accuracy and precision of the measurements. Mn(II) measurements were contrasted against standards of known concentration and blank samples. The analyses were carried out in triplicate, and Mn(II) and pH measurements were verified by performing triplicate readings. Furthermore, the measured Mn(II) concentrations were contrasted with measurements of random samples by ICP-OES.

## 3. Results

### 3.1. Adsorbent Characterization

#### 3.1.1. Fourier Transform Infrared (FT-IR) Spectroscopy

**Figure 1:**
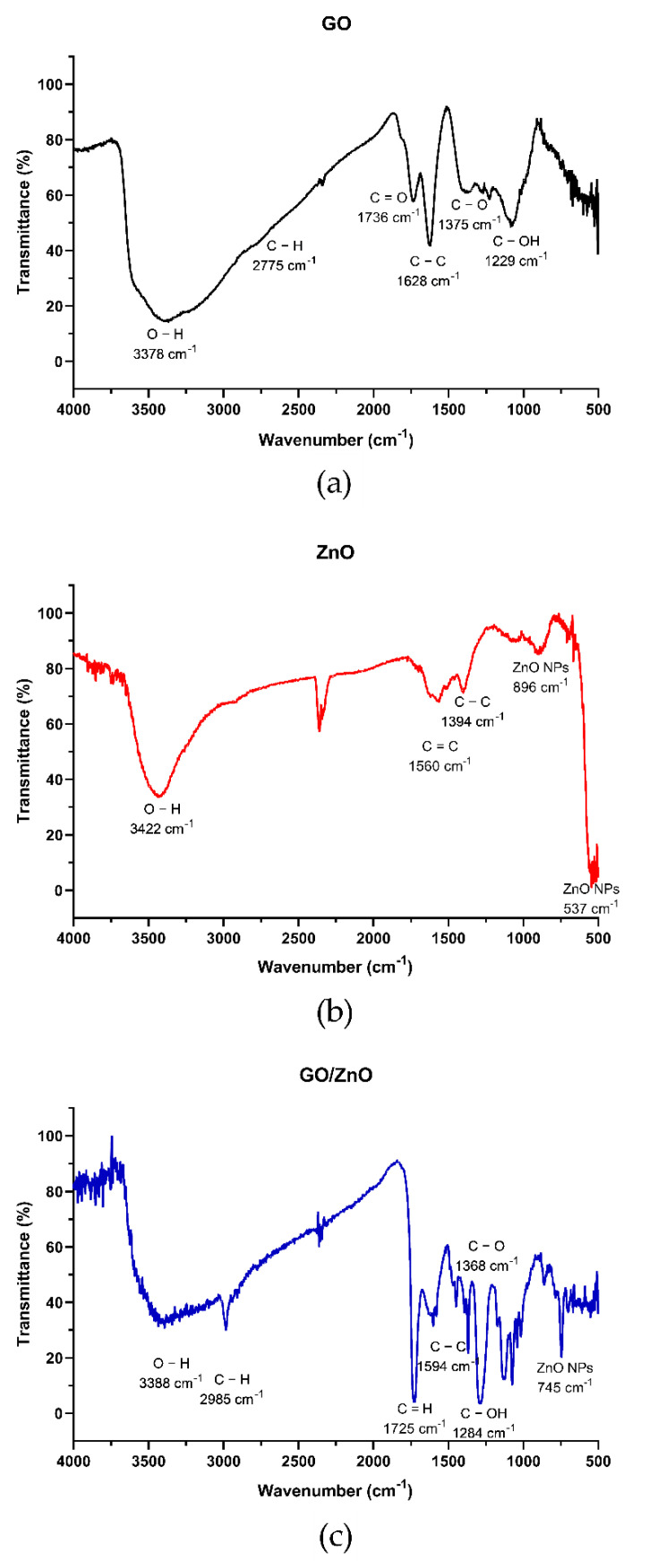
FT-IR spectrum of GO (**a**), ZnO (**b**) and GO/ZnO (**c**) nanocomposites before Mn(II) adsorption.

The chemical structure of the nanoadsorbents used in this study was characterized by Fourier Transform Infrared (FT-IR) spectroscopy. The FTIR spectra of GO, ZnO and GO/ZnO are shown in Figure 1. Figure 1a shows that the band corresponding to the hydroxyl group (O–H stretching, 3378 cm^−1^) is quite marked, which indicates the presence of these functional groups on the GO sheets [52]. In the same way, this band is also observed in GO/ZnO nanocomposites (Figure 1c). On the other hand, other absorption bands are observed at 1375, 1229 and 1081 cm^−1^, which correspond to C–H groups and C–O stretching [53,54], although these are less prominent than those corresponding to O–H groups. Functional groups of carboxylic acids, ketones, aldehydes or esters may be associated with the signal shown for carbonyl [55]. The absorption peak at 1375 cm^−1^ corresponds to the C–O stretching of the epoxide groups, while at 1229 cm^−1^, the stretching of the C–OH groups appears. Finally, the signal at 1081 cm^−1^ corresponds to the stretching of the C–O groups [53,54].

The FT-IR spectrum of the ZnO nanoparticles shows a broad band at 3422 cm^−1^ [56], which corresponds to the stretch vibration absorption band of water molecules in ZnO. The characteristic bands of melic oxides are also observed, which are below 1000 cm^−1^, specifically at 893 and 537cm^−1^ [57]. In addition, dispersion zones produced by the OH groups on the surface of the ZnO nanoparticles and hydrogen atoms that tend to form hydrogen bridges are observed [58,59]. 

In the GO/ZnO spectrum, the absorption peaks at 3388 cm^−1^ reflect the presence of OH groups characteristic of GO nanosheets. However, it is observed that these peaks are less thick, which is a consequence of the decoration of the GO nanosheets with the ZnO nanoparticles [60]. In the same way, it is observed that possibly the ZnO nanoparticles interact with the C=O groups, which is evidenced by the decrease in the adsorption peak at 1700 cm^−1^ [60]. Signals are observed at 1594 and 1368 cm^−1^ which correspond to the stretching of the C–C and C–OH groups, respectively [60]. In addition, the presence of the characteristic peaks of the ZnO nanoparticles is observed below 1000 cm^−1^ [57]. In this case, the signals at 745 and 435 cm^−1^ are clearly distinguished [60]. These results indicate that the decoration of GO with ZnO was effective and that the formation of the GO/ZnO nanocomposite occurs.

#### 3.1.2. Brunauer–Emmett–Teller (BET) Analysis

The Brunauer–Emmett–Teller (BET) surface areas of the GO and GO/ZnO nanocomposites were estimated by using N_2_ adsorption–desorption isotherms. The BET surface areas measured were 25.06 and 4.19 m^2^/g, for GO and GO/ZnO, respectively, which shows a reduction in surface area after decoration with ZnO nanoparticles. This smaller surface area may be a consequence of the lower dispersibility in water of the GO nanosheets compared to the smaller ZnO nanoparticles. Furthermore, the presence of the ZnO nanoparticles can block some of the GO pores, reducing the surface area [61]. This can also be observed by the reduction in pore volume that decreases from 0.07 cm^3^/g in the Go nanosheets to 0.01 cm^3^/g in the GO/ZnO nanocomposites. The pore density (15.06 nm for GO and 7.21 nm for GO/ZnO) also decreases with the decoration of ZnO nanoparticles. Despite this, the adsorption processes could be improved by the presence of ZnO nanoparticles in the GO nanosheets, independent of the reduction in the BET area, since the adsorption process also depends on other parameters.

#### 3.1.3. Scanning Electron Microscopy (SEM)

**Figure 2:**
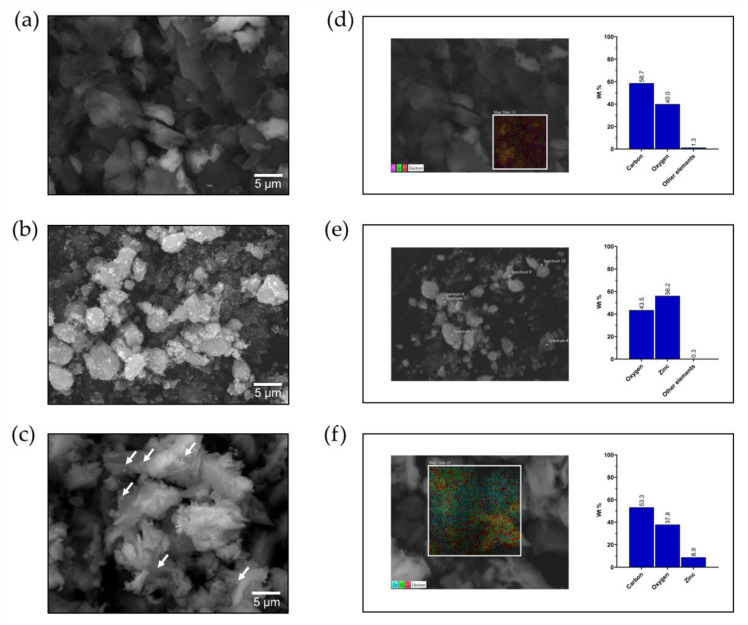
Scanning electron micrographs of GO (**a**), ZnO (**b**) and GO/ZnO nanocomposites (arrows show ZnO nanoparticles on GO nanosheets) (**c**). Elemental mapping of representative EDX spectrum of GO (**d**), ZnO (**e**) and GO/ZnO nanocomposites (**f**). For EDS analysis of ZnO nanoparticles, point scan mode was used in several points of the sample (Figure 2e).

Representative scanning electron microscopy (SEM) images of each nanoadsorbent are shown in Figure 2. GO layers (Figure 2a) differ from the typical straight sheets of graphite; this occurs by acid treatment using the Hummers method. Furthermore, Figure 2b shows the ZnO nanoparticles (<100 nm), which are shown to be aggregated into larger particles. Figure 2c shows that the ZnO nanoparticles (arrows) are scattered across the surface of graphene layers, in some cases with a characteristic aggregation of the interactions between ZnO particles. The decoration process with ZnO nanoparticles is characteristic of a crosslinking mechanism [41], and the morphology of GO/ZnO nanocomposites is uniform and defined.

EDX analysis was performed to determine the surface chemical composition of each nanoadsorbent. The results show that the GO nanosheets are mainly composed of carbon (58.7% wt) and oxygen (40% wt) with a low percentage of impurities (Figure 2d), while the ZnO nanoparticles are mainly composed of zinc (56.2% wt) and oxygen (43.5% wt) (Figure 2e). As expected, the EDX spectrum of the GO/ZnO nanocomposite shows the significant presence of carbon (53.3% wt), oxygen (37.8% wt) and zinc (8.9% wt) elements (Figure 2f). These results indicate that there is a lower proportion of ZnO nanoparticles compared to GO nanosheets in the nanocomposite, which is consistent with other studies that have shown a similar distribution of elements in the decoration of GO with ZnO nanoparticles [60].

### 3.2. Adsorption Experiments

**Figure 3:**
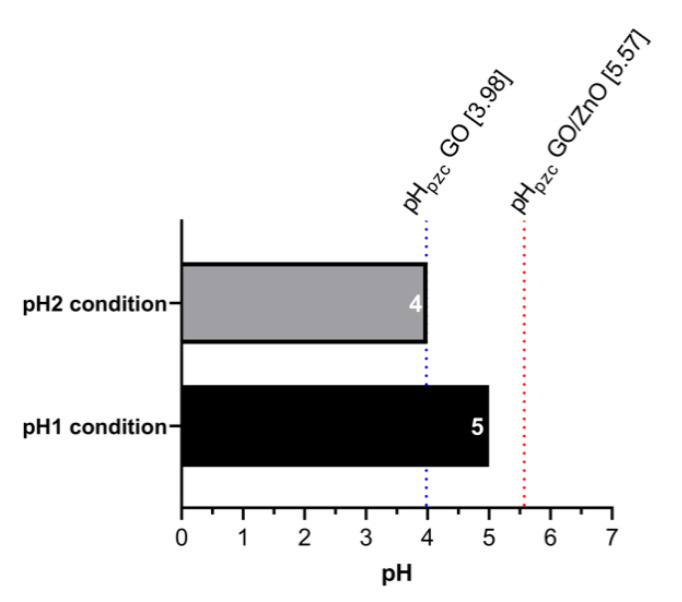
The pH values for Mn(II) solutions for experiments under pH1 and pH2 conditions. The blue line indicates the pH_PZC_ for GO and the red line for GO/ZnO nanocomposites.

Mn(II) adsorption experiments with nanoadsorbents (GO and GO/ZnO) were performed under two different pH conditions (pH1 = 5.0 and pH2 = 4.0) to determine the impact of acidic conditions on Mn(II) removal efficiency. Both conditions are typical of AMD waters in Northern Chile and represent two probable treatment scenarios for Mn(II)-enriched waters. To assess the impact of this parameter on the Mn(II) removal rates, the pH_PZC_ was calculated for each nanoadsorbent and compared with the prevailing pH conditions in the adsorption experiments. To assess the impact of this parameter on the Mn removal rates, the pH_PZC_ for each nanoadsorbent was calculated, comparing these with the prevailing pH conditions in the adsorption experiments. Figure 3 shows the pH values used in each experimental condition and the pH_PZC_ values of the materials used as nanoadsorbents in this study. 

For pH1 condition (pH1 = 5), it is observed that this value is lower than the pH_PZC_ of the GO/ZnO nanocomposites (pH_PZC_ = 5.57) and higher than the pH_PZC_ of GO (pH_PZC_ = 3.98), while under pH2 condition (pH2 = 4.0), it is observed that both GO and the GO/ZnO nanocomposites have a pH_PZC_ higher than the pH of the aqueous solutions.

pH is a factor that can affect the adsorption rates of different ions. Thus, the pH_PZC_ indicates at which pH value the surface charge is neutral. pH values higher than the pH_PZC_ determine that the surface charge of the adsorbate is negative, and consequently, positively charged ions can be more efficiently adsorbed [62]. The inverse effect is observed at pH < pH_PZC_, where the surface is positively charged and there is a greater repulsion effect with metal ions. The pH_PZC_ values for GO and GO/ZnO reported in this study are in the range observed by other investigations for GO (3.1–4.0) and GO/ZnO nanocomposites (6.0) [63,64,65]. Therefore, it was expected that the absorption of Mn(II) would not be theoretically efficient under two pH conditions (pH < pH_PZC_). Indeed, in both pH conditions, the adsorption of Mn(II) ions with GO is favored, and not with GO/ZnO nancomposites. Nevertheless, in the case of GO/ZnO nanocomposites, surface adsorption depends on other variables, such as the degree of functionalization, the impurities present and the efficiency of decoration with ZnO nanoparticles. In addition, the adsorption process of metallic ions such as Mn(II) does not depend solely on these factors but also depends on other variables, such as surface area of nanoadsorbents, surface chemical reactivity, porosity, the chemistry of the aqueous solution and the presence of competitors.

### 3.3. Adsorption Experiments under Different pH Conditions

**Figure 4:**
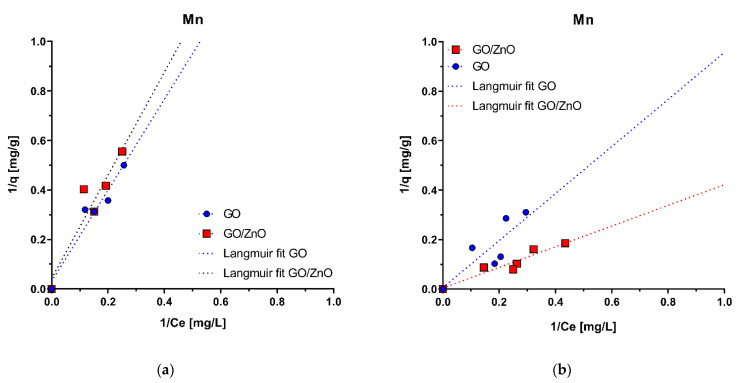
Adsorption isotherms for Mn(II) under pH1 condition (pH1 = 5.0) (**a**) and pH2 condition (pH2 = 4.0) (**b**) using GO and GO/ZnO nanocomposites as nanoadsorbent. The fit of linear form of the Langmuir-2 isotherm model is shown.

**Figure 5:**
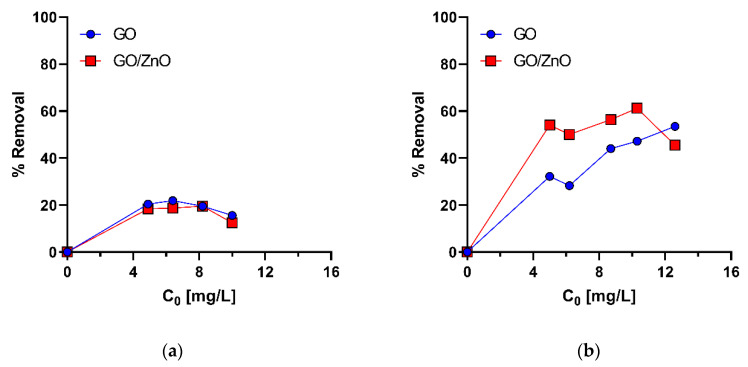
Removal percentages for Mn(II) under pH1 condition (pH1 = 5.0) (**a**) and pH2 condition (pH2 = 4.0) (**b**) using GO and GO/ZnO nanocomposites as nanoadsorbents. C_0_ is the concentration of Mn(II) ions at the initial time.

Experimental results of Mn(II) adsorption are shown in Figure 4. Under two pH conditions (pH1 = 5.0 and pH2 = 4.0), the pH value of Mn(II) solutions could more efficiently favor the adsorption of Mn(II) on GO compared with GO/ZnO, according to the surface charge of the nanoadsorbents (pH_PZC_). However, the results do not show significant differences between the adsorption of Mn(II) with GO and GO/ZnO nanocomposites at pH = 5.0 (Figure 5a). On the contrary, under the pH2 condition (pH = 4.0), it is observed that the Mn(II) removal rates are more efficient with GO/ZnO than GO (Figure 5b), which suggests that the adsorption process is relatively independent of the pH_PZC_ of the nanoadsorbents. Yan et al. [66] previously showed that there were no marked differences in the removal of Mn(II) ions with GO between pHs 4 and 5. However, we observed differences in Mn(II) adsorption with GO/ZnO between both pHs. Thus, our results show that the removal of Mn(II) ions is more efficient using these nanocomposites compared to GO, which may be a consequence of the decoration with ZnO nanoparticles. Previously, our research group observed similar results for the removal of Cu(II) and Al(III) ions from acidic waters, where GO/ZnO showed better adsorption rates compared to GO [44].

The fit to the experimental data shows that the Langmuir and Freundlich isotherm models have the best fit in both pH conditions (Table 2). The determination coefficients (R^2^) of the linear forms for the Langmuir and Freundlich models were both high compared to a worse fit of the Tempkin model. In the case of the Langmuir model, it is assumed that the adsorption mechanism occurs in a monolayer and, therefore, a finite number of ions will be adsorbed on the surface [46], while in the Freundlich model, reversible heterogeneous adsorption is described without restricting the adsorption process of the formation of a monolayer [47]. For this reason, the Freundlich isotherm predicts that the adsorbate concentration on the adsorbent will increase without saturation according to how the adsorbate concentration increases in the aqueous solution [67]. The similar fit of both models suggests that both mechanisms are involved in the removal of Mn(II) with GO and GO/ZnO nanocomposites. 

The adsorption of Mn(II) ions can occur by the formation of a monolayer on the surface of the nanoadsorbents independent of the pH value and followed the Langmuir model. However, the heterogeneity of functional groups on the surface of GO can also favor different interactions between adsorbate and adsorbent, resulting in a Freundlich model adsorption mechanism. Yan et al. [66] obtained similar adjustments for Mn removal from micropolluted water using magnetic GO. They obtained good fits for Langmuir (R^2^ = 0.9480) and for Freundlich (R^2^ = 0.9722). On the other hand, the high surface area of ZnO nanoparticles given by their nanometric scale makes them good candidates for adsorbing positive metal ions from aqueous solutions [68,69,70,71]. 

The q_m_ values of the Langmuir model for the pH1 condition (pH = 5.0) are 3.2 × 10^1^ mg/g and 2.3 × 10^1^ mg/g for the adsorption with GO and GO/ZnO nanocomposites, respectively. On the other hand, for the pH2 condition (pH = 4.0), the q_m_ values are higher, reaching values of 1.8 × 10^2^ mg/g and 2.6 × 10^2^ mg/g for GO and GO/ZnO, respectively, which strongly reflects the better Mn(II) removal performance of GO/ZnO nanocomposites at pH = 4.0 (Table 2). For the fit with the Freundlich model, the K_f_ values are similar between GO and GO/ZnO nanocomposites, although these values are also slightly higher for the pH2 condition (pH = 4.0), supporting the behavior observed in the experimental data (Figure 4).

Figure 5 shows the removal rates of Mn(II) ions for each adsorbent under two pH conditions. As in the adsorption isotherms, a higher removal rate is observed under the pH2 condition (pH2 = 4.0) for both nanoadsorbents, reaching maximum removal rates of 61.2% for GO/ZnO nanocomposites (C_0_ = 10.3 mg/L) and 53.5% for GO (C_0_ = 12.6 mg/L). In this case (pH2 = 4.0), both nanoadsorbents have a lower pH_PZC_ than the pH of the solution, so it was expected that the removal would be more efficient, which is confirmed by comparing the removal of Mn(II) in both conditions. Furthermore, the effect of pH is also evident when comparing the increase in Mn(II) removal with GO between pH1 = 5.0 and pH2 = 4.0. It is observed the removal was greater at pH = 4.0, which was expected, since this pH < pH_PZC_ and, therefore, the effect of attraction of positive ions is greater when the surface is negatively charged; the inverse effect is seen at pH = 5.0, where the surface is positively charged. On the other hand, the higher removal efficiency of Mn(II) ions with GO/ZnO nanocomposites under the pH1 condition (pH = 4.0) (Figure 5a) can also be sustained by a higher amount of negative charges on the surface of the nanocomposite compared to the pH1 condition (pH1 = 5.0). Thus, the efficiency of attraction of Mn(II) ions by this surface is much higher and could explain the differences in removal rates between GO and GO/ZnO observed at this pH, which are not evident under the pH1 condition (pH1 = 5) (Figure 5a). Although there are few studies evaluating Mn(II) removal with GO/ZnO nanocomposites, the Mn(II) removal rates reported in this study were similar to some removal rates reported with similar nanomaterials. For example, Yang et al. [39] found that between pH 4.0 and 7.0, the adsorption efficiency of Mn(II) by Sodium Alginate/GO Composite remains almost unchanged and reaches removal rates of ~55%. Additionally, Xu et al. [40] reported that poly (sodium acrylate)-graphene oxide (PSA-GO) double network hydrogel adsorbent achieves Mn(II) removal rates of 84% when the pH value is above 3.0. This supports and confirms our results. Le et al. [70] found that the removal of Mn(II) ions from aqueous solutions by ZnO nanoparticles is mainly mediated by mechanisms of absorption of Mn(II) ions onto the negatively charged surface of ZnO particles rather than by photocatalytic reaction of ZnO under UV irradiation. They indicate that under visible light conditions, the reduction potential standard of Mn^2+/^Mn (E^0^ = −1.18 V vs. NHE) turns out to be more negative than the e–CB level of ZnO particles [45], and, therefore, the oxidation pathway of Mn(II) to Mn(IV) would be the most viable alternative for the removal of Mn(II) ions. However, the results of Mn(II) removal under the pH2 condition (pH = 4.0) suggests that the combined surface adsorption mechanisms between ZnO nanoparticles and the interaction with functional groups on the surface of GO are more likely to explain the Mn(II) removal rates observed for GO/ZnO nanocomposites. Thus, from these data, it is possible to infer that the pH = 4 increases the efficiency of the removal of Mn(II) ions by both nanoadsorbents, but this is much better with GO/ZnO nanocomposites. However, the differences in the removal rates, as mentioned above, may also depend on other factors, such as the type of adsorbent used, the surface functional groups, the surface properties, the pH_PZC_ values, the decoration efficiency and availability of ZnO nanoparticules on the surface of GO.

On the other hand, along with the adsorption capacity, the desorption process is also important because the reusability and regeneration of the adsorbent have a direct impact on the operating costs of the technologies based on these processes. For most adsorbents, the adsorption properties are not recoverable after the adsorption process [72]. Although the use of nanomaterials can improve the adsorption capacity of various pollutants, it is important to develop new reusable nanoadsorbents. Wang et al. [72] found that reduced GO/ZnO composite shows a high cycling performance for Rhodamine B with adsorption up to 99% over four cycles. Likewise, Qiao et al. [73] demonstrated that magnetic GO/ZnO nanocomposites maintain more than 80% of the initial adsorption capacity of tetracycline contaminants after four cycles of regeneration. These studies support the idea that GO/ZnO nanocomposites have high potential as a recyclable adsorbent for water treatment, which can reduce operating costs in practical applications.

### 3.4. Kinetic Studies of the Adsorption

**Figure 6:**
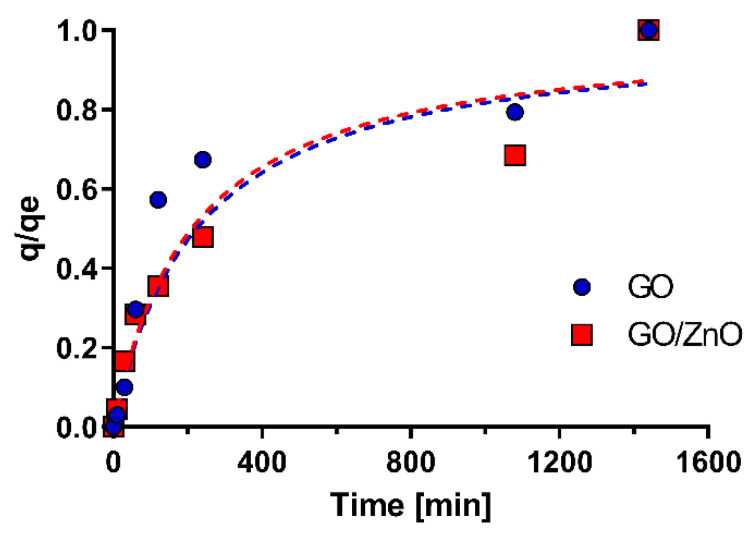
Effect of contact time on removal Mn(II) ions. Kinetic curves for Mn(II) are based on the pseudo-second-order model.

Contact time is a critical parameter for the adsorption process; therefore, it is necessary to determine the kinetics of the adsorption of GO and GO/ZnO nanocomposites. The removal of Mn(II) ions by GO and GO/ZnO nanocomposites was investigated at an initial concentration of Mn of 28.4 mg/L, pH = 4.0 and a constant ZnO and GO/ZnO dosage for different time intervals. Figure 6 shows the variation in the adsorption capacity trends versus contact time. A removal of 40% for GO and 25% for GO/ZnO is observed at 2 h, which indicates that the initial removal with GO is faster than with GO/ZnO nanocomposites. However, at 18 h, the removal of Mn by GO increases by 55% and 48% for GO/ZnO, while at 24 h the percentage is similar (~70%) for both nanoadsorbents. Thus, after 18h, the adsorption capacity gradually reaches equilibrium. Yang et al. [39] showed that the removal of Mn(II) by Sodium Alginate/Graphene Oxide Composite reaches equilibrium after 210 min, showing faster reaction kinetics compared to our results. Meanwhile, Yan et al. [66] showed that magnetic GO had a shorter equilibrium time during the adsorption of both Fe and Mn, reached in 15 min, which supports that these nanomaterials have a rapid adsorption efficiency. Kinetic studies performed with ZnO nanoparticles for the removal of metals, such as lead, cadmium or copper, have shown equilibrium times of 100 min or less [74,75]. In our case, the kinetics were slower for both GO and GO/ZnO, which is possibly influenced by the initial concentration of Mn(II) and the inherent properties of the nanoadsorbents used.

To examine the adsorption mechanisms involved in the removal of Mn(II), pseudo-first and pseudo-second-order kinetic models were applied to the experimental data. Table 3 shows the kinetic adsorption parameters for both models. From the analysis, it appears that the R^2^ for the PSO model fits better to the experimental data with values of 0.9606 and 0.9382 for GO and GO/ZnO, respectively. Likewise, the values of q_e2_ were greater than the values of q_e1_, confirming that this model presents a better fit compared with the PFO model. These results are supported by the literature, where it has been shown that the pseudo-second-order kinetic model represents better data on the adsorption kinetics of heavy metals [32,51,76,77,78,79]. The diffusion-based mechanisms mainly explain the adsorption process described by the PSO model [51,76]. In addition, this model adequately explains the different affinities between adsorbent and adsorbate derived from the properties of the ZnO nanoparticles and the functional groups present in the GO nanosheets. Therefore, the results of the adsorption kinetics are consistent and support the results of the Mn(II) adsorption isotherms.

Additionally, to determine if the Mn(II) adsorption process occurs by chemical or physical adsorption, the Mn(II) adsorption rates were compared with the changes in the specific surface area of the nanoadsorbents [80]. It can be seen that the BET surface area decreases with the decoration with ZnO nanoparticles. Indeed, a decrease in the BET surface 83% (25.06 and 4.19 m^2^/g for GO/ZnO) is observed. In contrast, Mn(II) removal rates at pH2 = 4.0 increase for GO/ZnO nanocomposites compared to non-functionalized GO, which indicates that the adsorption of Mn(II) is primarily a chemical adsorption process. This is also supported by the comparison with the pH_PZC_ value at pH2 = 4.0, where electrostatic attraction is a key factor for Mn(II) removal and significantly influences adsorption capacity.

To complement the analysis of the kinetic models, the approaching equilibrium factor (Rw) was calculated for each kinetic study. This factor is useful to describe the type of kinetic curve and the proximity to the equilibrium of the studied system. In this way, with this analysis, it is possible to determine the efficiency of the adsorption process [51]. Thus, the characteristic adsorption curve is called approaching equilibrium if the range is in 1 > Rw > 0.1, which can be observed for both nanoadsorbents (GO and GO/ZnO). The parameters of the Rw factor in the PSO kinetic model are shown in Table 4. From the analysis, it appears that the curves for both GO and GO/ZnO are very close to equilibrium for the evaluated period (24h), which supports a good performance and the reliability of the results in the equilibrium time used for the adhesion isotherms (20 h).

## 4. Conclusions

The results of this study show that GO decorated with ZnO nanoparticles shows a higher removal efficiency of Mn(II) ions from acidic waters compared with non-functionalized GO. ZnO nanoparticles were observed on the surface of GO nanosheets through SEM-EDX analysis. Furthermore, FTIR results show that the decoration process was effective. The pH_PZC_ of GO/ZnO nanocomposites was 5.57, which is quite high for acidic water treatment. On the contrary, non-functionalized GO has a lower pH_PZC_ (3.98), which makes it theoretically more efficient for the removal of heavy metals. However, the results showed that the removal rates of Mn(II) were similar for both nanoadsorbents (GO and GO/ZnO) at pH = 5.0 and significantly higher for GO/ZnO nanocomposites at pH = 4.0. This indicates that the functionalization with ZnO nanoparticles considerably improves the adsorption capacity of Mn(II), which, even under acidic conditions, is theoretically less favorable. The presence of ZnO nanoparticles on the surface of GO improves the adsorption of Mn(II) under more acidic conditions, which is also evidenced by the fact that the removal rate of Mn(II) ions with GO does not change between both pH conditions (pH1 = 5.0 and pH2 = 4.0), while the decoration with ZnO favors the removal at more acidic pH (pH2 = 4.0) with GO/ZnO nanocomposites. On the other hand, the pH 2 condition (pH2 = 4.0) is very close to the pH_PZC_ of non-functionalized GO (3.98), which suggests that the removal efficiency of Mn(II) ions is related to this value, and consequently, the pH can be a critical factor in increasing the adsorption rates of GO/ZnO nanocomposites. The Mn(II) adsorption process fits both with Langmuir and Freundlich models, suggesting that both mechanisms are involved in the removal of Mn(II) ions with GO and GO/ZnO nanocomposites. Thus, adsorption of Mn(II) ions can occur by the formation of a monolayer on the surface of the nanoadsorbents and by interactions with functional groups on the surface of GO and ZnO nanoparticles, resulting in a Freundlich model adsorption mechanism. Additionally, adsorption isotherms were efficiently modeled with the pseudo-second-order kinetic model.

These findings contribute to understanding the adsorption mechanisms of Mn(II) in GO/ZnO nanocomposites and how functionalization with ZnO nanoparticles can significantly improve the adsorption under acidic conditions. Furthermore, this research is the first to study the application of GO/ZnO nanocomposites for the treatment of Mn(II)-enriched AMD waters. However, future efforts are necessary to determine other critical variables of the adsorption process with GO/ZnO nanocomposites under more complex and realistic conditions, such as multimetallic waters and with the presence of competitors. Furthermore, given the complexity of the nanomaterials used, it is necessary to study the recovery of the materials in order to evaluate their potential for scaling up to real acid water treatment systems.

## Figures and Tables

**Table 1:** List of adsorption isotherms models used in this study.

Isotherm	Nonlinear Form	Linear Form	Plot	Variables	Reference
Langmuir-2	$q_{e}=\frac{q_{m}K_{l}C_{e}}{1+K_{L}C_{e}}$	$\frac{C_{e}}{q_{e}}=\frac{C_{e}}{q_{m}}+\frac{1}{\left(q_{m}K_{L}\;\right)}$	$\frac{1}{q_{e}}vs.\frac{1}{C_{e}}$	$K_{L}=\frac{Intercept}{Slope}$ $q_{m}=Intercept^{-1}$	[46]
Freundlich	$q_{e}=K_{F}C_{e}^{\frac{1}{n}}$	$log\left(q_{e}\right)=log\left(K_{F}\right)+\frac{1}{n\;log\left(C_{e}\right)}$	$log\;\left(q_{e}\right)\;vs.\;log\;\left(C_{e}\right)$	$K_{F}=antilog\left(intercept\right)$ $n=\frac{1}{Slope}$	[47]
Tempkin	$q_{e}=B_{T}In(K_{T}C_{e})$	$q_{e}=B_{T}InK_{T}+B_{T}InC_{e}$	$q_{e}\;vs.\;In\left(C_{e}\right)$	$K_{T}=exp\left(\frac{Intercept}{BT}\right)$ BT=Slope	[48]

**Table 2:** Parameters for the Langmuir, Freundlich and Tempkin isotherm models for Mn(II) adsorption performed under two pH conditions.

		Langmuir			Freundlich				Tempkin	
pH Condition	Nanoadsorbent	$q_{m}\;(\mathrm{mg}/g)$	$K_{L}\;(L/\mathrm{mg})$	R^2^	$K_{F}\;(L/g)$	n	R^2^	$K_{T}\;(L/g)$	$B_{T}\;(\mathrm{mg}/L)$	R^2^
5.0	GO	3.2 × 10^1^	1.7 × 10^−2^	9.4 × 10^−1^	1.0 × 10^0^	1.7 × 10^0^	9.7 × 10^−1^	1.0 × 10^0^	1.6 × 10^0^	9.7 × 10^−1^
	GO/ZnO	2.3 × 10^1^	2.0 × 10^−2^	8.9 × 10^−1^	1.0 x10^0^	2.0 × 10^0^	8.8 × 10^−1^	1.0 × 10^0^	1.4 × 10^0^	8.9 × 10^−1^
4.0	GO	1.8 × 10^2^	6.0 × 10^−3^	7.1 × 10^−1^	1.1 × 10^0^	1.1 × 10^0^	7.6 × 10^−1^	1.1 × 10^0^	3.5 × 10^0^	5.7 × 10^−1^
	GO/ZnO	2.6 × 10^2^	9.0 × 10^−3^	9.1 × 10^−1^	1.3 × 10^0^	7.4 × 10^−1^	8.8 × 10^−1^	1.0 × 10^0^	6.7 × 10^0^	8.6 × 10^−1^

**Table 3:** Kinetic adsorption parameters for pseudo-first-order and pseudo-second-order models.

Nanoadsorbent	$q_{e}^{exp}\;(\mathrm{mg}/g)$	Pseudo-First-Order			Pseudo-Second-Order		
		$k_{1}\;(1/\min)$	$q_{e1}\;(\mathrm{mg}/g)$	*R* ^2^	$k_{2}\;(g/\mathrm{mg}\;\min)$	$q_{e2}\;(\mathrm{mg}/g)$	*R* ^2^
GO	4.0 × 10^1^	1.2 × 10^−3^	2.8 × 10^1^	7.1 × 10^−1^	1.0 × 10^−4^	4.3 × 10^1^	9.6 × 10^−1^
GO/ZnO	4.0 × 10^1^	9.2 × 10^−4^	3.2 × 10^1^	8.7 × 10^−1^	1.2 × 10^−4^	4.0 × 10^1^	9.4 × 10^−1^

**Table 4:** The approaching equilibrium factor (Rw) in the pseudo-second-order kinetic model.

Nanoadsorbent	$q_{e}\;(\mathrm{mg}/g)$	$k_{2}\;(g/\mathrm{mg}\;\min)$	$t_{ref}\;(\min)$	$R_{w}$	Type of Kinetic Curve	Approaching Equilibrium Level
GO	4.3 × 10^1^	1.0 × 10^−4^	1440	1.3 × 10^−1^	Slightly curved	Approaching equilibrium
GO/ZnO	4.0 × 10^1^	1.2 × 10^−4^	1440	1.3 × 10^−1^	Slightly curved	Approaching equilibrium

## Data Availability

The data presented in this study are available on request from the corresponding author.

## Sample Availability

Samples of the compounds are not available from the authors.
